# Microbial Inoculation for Productivity Improvements and Potential Biological Control in Sugar Beet Crops

**DOI:** 10.3389/fpls.2020.604898

**Published:** 2020-12-22

**Authors:** Gonzalo Sacristán-Pérez-Minayo, Domingo Javier López-Robles, Carlos Rad, Luis Miranda-Barroso

**Affiliations:** ^1^Microbiology Section, Faculty of Sciences, University of Burgos, Burgos, Spain; ^2^Edaphology and Agricultural Sciences Section, Faculty of Sciences, University of Burgos, Burgos, Spain; ^3^Sustainable Agriculture Department, Syngenta-Spain, Madrid, Spain

**Keywords:** integrated crop management, PGPR, sugar beet, photosynthesis parameters, sucrose

## Abstract

Used mainly for sucrose production, sugar beet is one of the most important crops in Castilla y León (Spain). Several studies have demonstrated the benefits of microorganisms in different crop management programs, among which Plant Growth Promoting Rhizobacteria (PGPR). This research aims to assess the beneficial effects of two PGPRs strains (*Pseudomonas fluorescens* Pf0-1 and *Pseudomonas chlororaphis* CECT 462) on sugar beet (*Beta vulgaris*) production. Three treatments: a PGPRs co-inoculation assay of untreated seeds without any chemical treatment (TB), a conventional treatment with commercial seeds and fungicide application (TT); and a control with seeds without protective coating, bacterial inoculation and chemical treatment (ST). The efficacy of PGPRs inoculation on sugar beet production was determined measuring periodically the photosynthetic status of plants, and the final yield and quality of tubers. Aerial and root plant biomass, maximum beet perimeter, polarization, and sugar values of the sugar beet plants inoculated with PGPRs showed higher values and significant differences to sugar beet subjected to other treatments. We could see that PGPRs inoculation (TB treatment) produced significant differences in the quantum yield of PSII (ΦPSII). TB showed the highest value for ΦPSII and the NPQ (non-photochemical quenching), the lowest value, even though the PSII (maximum quantum yield of photosystem II) was very similar in all treatments. The two assayed PGPR strains triggered a significant increase in sugar beet production yield and quality. PGPRs inoculation techniques could be used in different crops and they could be applied as biofertilizers, improving the agricultural production.

## Introduction

Sugar beet (*Beta vulgaris* L. var. saccharifera) is an important root crop in moderate climates and the main source of sugar (Dohm et al., 2014). The worldwide cropping area covers over approximately 4.5 million Ha, with roughly 70% of sugar beet production in Europe (FAOSTAT, 2019). Annual world sucrose production stands 175.6 million tons in 2017, of which 28% is extracted from sugar beet (*Beta vulgaris* L.), and the remainder from sugar cane OECD/FAO (2020). Sugar production in 2018 was approximately 2,870,907 tons (FAOSTAT, 2019). In Spain, sugar beet cultivation is reported to cover about 53,000 Ha. The present research was conducted in the region of Castilla y León, where sugar beet is one of the most important industrial crops, providing over 50% of all Spanish beet sugar. Sugar beet crop profitability is valued in terms of sugar production, which basically refers to its sucrose purity factor, as sucrose content is made up of more than 99.5% in the final white crystalline sugar (Pan et al., 2015).

Several research studies have noted the importance of soil microbiome on plant health, in particular in sugar beet crops (Berendsen et al., 2012; Bakker et al., 2013, 2020; Weijuan et al., 2020; Wolfgang et al., 2020). In fact because the genome and breeding history is known, sugar beet is an interesting model crop for microbiome studies (Zachow et al., 2008; Mendes et al., 2011; Würschum et al., 2013; Dohm et al., 2014; Kusstatscher et al., 2019a, b). Hiltner (1904) established the importance of the rhizosphere microbiome for growth promotion in crops and omics technologies allow in-depth analysis, nowadays (Mendes et al., 2011; Raaijmakers and Mazzola, 2016).

Bacteria with multiple beneficial traits can be advantageous in commercial agriculture and are relevant to the bio-economy (Backer et al., 2018). Recently, research on Plant Growth Promoting Rhizobacteria (PGPR) for crop improvements are gaining prominence and thousands of research works have been published so far (Compant et al., 2005; Mia et al., 2010; Backer et al., 2018). The term PGPR was first defined by Kloepper et al. (1980) in the 1980s, later Compant et al. (2005) subsequently described PGPR as microorganisms from the rhizosphere that can positively influence plant growth and plant health. These PGPRs have ability to protect the plants from drought, salts and heavy metal stresses and play significant role in the plant growth promotion, yields, nutrient acquisition and as well as minimizing the use of chemical fertilizers (Kumar et al., 2019). In particular, PGPRs could promote directly plant growth by various mechanisms, including: (i) the production of phytohormomes such as auxins, cytokinins and gibberellins (Santner et al., 2009); (ii) the production of plant growth promoting (PGP) substances such as indole-3-acetic acid (IAA) and/or siderophores which can provide soluble iron to plants (Scher and Baker, 1982); (iii) the increase of the solubilization of phosphorus and other trace element for plant uptake (Gyaneshwar et al., 2002); (iv) the supply of nutrients to plants, by asymbiotic nitrogen fixation (Doberein and Campelo, 1971) and v) the secretion of enzymes that can modulate plant growth and development, such as reducing ethylene level by synthesis of 1-aminocyclopropane-1-carboxylic acid (ACC) (Yang and Hoffman, 1984; Penrose et al., 2001). The use of PGPRs as biological control agents have been found effective and are being increasingly applied in the field (Pooja et al., 2019). Indirectly, some PGPRs are able to reduce the disease development in plant system by various mechanisms that include among others: production of antimicrobial metabolites, volatile compounds and induced systemic resistance (ISR) (Hassani et al., 2018; Stringlis et al., 2018).

Sugar beet is known to be affected by several pathogens, including bacteria, fungi, viruses and nematodes. *Cercospora beticola* Sacc. and *Erysiphe betae* (Vañha) Weltz are the causative agents of Cercospora Leaf Spot (CLS) and powdery mildew, respectively, and they are the most damaging foliar diseases for sugar beet crops (Jay et al., 2020). CLS occurs in sugar beet-growing areas worldwide and can lead to reductions in recoverable sucrose of 30–48% (Khan et al., 2001). CLS is a polycyclic disease whose severity depends on weather conditions *C. beticola*’s full disease cycle can occur in as few as 10 days under suitable climatic conditions of high relative humidity and high temperatures, thus resulting in multiple infection cycles in each growing season. Fungus conidia infect leaves, resulting in the appearance of millimeter-scale brown round spots. These necrotic spots then expand and coalesce, eventually defoliating the whole plant and requiring new leaves to grow. Beet powdery mildew is also another worldwide fungal disease of sugar beet. Powdery mildew is characterized by a white dust-like mycelium that develops over the leaf surface. Heavily infected tissues develop chlorosis and suffer early senescence, with infection being especially damaging in areas with arid climates, such as in Mediterranean countries (Fernández-Aparicio et al., 2009). Fungal diseases incidence can occur on sugar beet in one season, consecutively or simultaneously, and outbreaks can result in a significant loss of the crop in years with high disease pressure (Heick et al., 2020). The severity and frequency of fungal disease attacks vary considerably every year, depending on factors as weather conditions, microclimate, and agronomic practices (Heick et al., 2020). The traditional treatment for these fungal diseases involves prevention, in other words treatment with highly fungicidal phytosanitary products may be effective in controlling the development of both pathogens incidence. However, a significant reduction of the use of fungicides is highly desired since, some of them, affect the environment while being expensive (Van Zwieten et al., 2004).

There is therefore a need for complementary agricultural practices, such as the use of microorganism-based biological control methods (Compant et al., 2005; Naureen et al., 2009). *Pseudomonas* and *Bacillus* are the most commonly described genera possessing plant growth promoting activities (Munees and Mohammad, 2011). *Pseudomonas* are frequently found across all life stages of sugar beet and that several strains with promising biocontrol potential were found in sugar beet endosphere (Zachow et al., 2008, 2010).

*Pseudomonas chlororaphis* is capable of promoting the growth of plants such as wheat and corn (Agaras et al., 2020) or millet (Niu et al., 2018). It has been possible to verify the protective capacity against different pathogens such as fungi (*Rhizoctonia solani*) where it induced the plant to increase the expression of certain genes that influenced its protection (Kamou et al., 2020). Indirectly, *Pseudomonas fluorescens* Pf0-1 stimulates plant growth by protecting it from attack by *Pythium ultimum*, *Gaeumannomyces graminis* var. *tritici*, and *Fusarium oxysporum*, in addition to the motility and degree of chemotaxis that it possesses, which are essential properties in the colonization of vegetable roots (Oku et al., 2012).

In the present work, we evaluated the successive application of a mixed PGPRs culture (*Pseudomonas fluorescens* Pf0-1 and *P. chlororaphis* CECT 462) on the productivity of sugar beet evaluated in terms of production yield and sucrose content, and physiological changes in the whole cycle of the plant. Additionally, we also monitored the biocontrol effects of PGPRs against two fungal pathogens, *Cercospora beticola* and *Erysiphe betae*, throughout the sugar beet production cycle.

## Materials and Methods

### Plant Material and Bacterial Strains

Seeds of *Beta vulgaris* var. Turbata, tolerant to the fungal pathogens *Cercospora beticola* and *Erysiphe betae*, were provided by Koipesol Semillas, S.A. These commercial seeds have a protective coating containing fungicide and insecticide formulations. The sugar beet standard planting density was 100,000 plants per hectare.

The present research was conducted in the region of Castile and Leon, where sugar beet is one of the most important industrial crops, providing over 50% of all Spanish beet sugar (Esteban Baselga, 1993). The present experiment was performed in the 2018 sugar beet campaign, from April to November.

In the present experiment, we applied a combination of two PGPRs strains, *Pseudomonas fluorescens* Pf0-1 and *Pseudomonas chlororaphis* CECT 462. The first of the *Pseudomonas* strains was isolated from an agricultural soil in Pampliega (42°12′N; 3°58′W; altitude 809 m asl), Burgos (Spain) (Sacristán Pérez-Minayo et al., 2011). We used the Fasta Nucleotide Similarity Search Database available from the European Bioinformatics Institute (EMBL-EBI)^1^ and we obtained a percentage of similarity of 100% and a percentage of identity of 99.107% compared with *Pseudomonas fluorescens* Pf0-1 (EMBL: CP000094). *Pseudomonas chlororaphis* CECT 462 was provided by the Spanish Collection of Type Cultures (CECT — Colección Española de Cultivos Tipo, 2020). To determine the compatibility between PGPR strains, a cross-streak method of inoculation was done checking the appearance of inhibition zones at the intersection of the paired strains (Santiago et al., 2017). The bacterial strains were maintained at −80°C in nutrient broth with 20% glycerol. Inocula were prepared, separately, by streaking strains at −80°C onto King A medium (Cultimed, Spain), incubating plates at 30°C for 24 h. After incubation, the plates were scrapped off into a sterile 10 mM SO_4_Mg buffer at a suspension of 10^8^ CFU/ml. This final suspension contained both PGPR strains and was prepared repeatedly before each PGPRs spraying applications.

### Experimental Design

The present experiment was performed in the 2018 sugar beet campaign, from 8th April to 26th November. The experimental plot occupied 252 m^2^ (18 m long and 14 m wide) of an irrigated field (32.30 Ha) located on the outskirts of Pedrosa del Rey, Valladolid. The site is placed at 706 m of altitude. Annual mean temperature was 12°C, minimum temperature (−0.3°C) was found in January and the maximum (29.4°C) in July. In relation with frost days, the last frost day of spring was 13rd April and first frost day of autumn was 8th November. Hence, we had 208 free frost days. The mean annual rainfall was 374 mm with 61 rainfall days per year (ITACYL — Instituto Tecnológico Agrario de Castilla y León, 2019). The field had corn as precedent crop and is classified as LVk Calcaric Luvisol (IUSS Working Group WRB, 2015). Main soil properties are: texture, pH, EC, SOM, Total N. The soil pH and the organic matter content were 8.3 and closely to 2%, respectively. The texture of the soil in the experimental plots was, in general, loamy, except some small area was found as loamy-clayey. The mean clay content was 25.65%. We performed three different treatments in a completely randomized block design: TB, without seed coating, with PGPRs inoculum application and chemical spraying; TT, with seed coating and chemical spraying and without PGPRs inoculum and ST, without seed coating, chemical spraying and PGPRs inoculum. The chemical spraying consisted of a mixture of “Karate King” insecticide (0.5 Kg/Ha), “Tilt” fungicide (1.25 l/Ha) and *boron-molybdenum* fertilizer (2.50 l/Ha). Each treatment had four replicates of 2 × 2 m^2^ subplots, in which 40 plants were introduced, and with 2 m interrow spaces between them, to avoid border effect. Irrigation was performed using a central pivot system (30–50 l/m^2^ per week) throughout the whole production cycle, according local irrigation schedule.

The seeds protective coating was removed in TB and ST treatment, with thorough washing and stirring of seeds in sterile distilled water, with the purpose of check the inoculum effect without the presence of fungicide or herbicides. After coating removing, the seeds in the TB trial were inoculated by immersion for 6 h at 30°C with the mixed culture of PGPR strains, *Pseudomonas fluorescens* Pf0-1 and *Pseudomonas chlororaphis* CECT 462. Immediately after sowing, the seeds for the TB replicates were irrigated once with the mixed PGPRs inoculum (1 ml per seed). The plant leaves in the TB replicates were sprayed six times with the PGPRs suspension (1,500 ml each sprayed time). Foliar spray application was performed to promote microorganism-plant interactions during the production cycle. The dates of the PGPRs spraying applications were: first PGPRs spraying, 23 May 2018; second PGPRs spraying, 19 June 2018; third PGPRs spraying, 01 July 2018; fourth PGPRs spraying, 31 July 2018; fifth PGPRs spraying, 02 September 2018, and sixth PGPRs spraying, 08 October 2018. The TT subplots were initially irrigated with water and thereafter, sprayed with water and a mixture of insecticide, fungicide and fertilizer, at the same time as the TB treatment. ST subplots were irrigated and sprayed with water six times. The timelines for the three treatments are shown schematically in Supplementary Figure 1.

### Sugar Beet: Crop Production Yield and Quality

At the end of the production cycle (232 days after sowing), 10 plants were harvested per subplot and the following parameters analyzed: sugar content (kg), polarization (%), corrected sucrose, N-amino, potassium (K) and sodium (Na) content and industrial loss and yield (%) according to the International Sugar Scale. The total, aerial and root biomass (kg) were also recorded, as well as the root maximum beet perimeter and length. Sucrose content was measured by polarization (Schmidt and Haensch Mod. 14220), Na and K content by flame photometry (Model NAK-1 Pacisa), and α-amino-nitrogen content (α-N) according to the Stanek and Pavlas (1934) blue index method, as modified by the Swedish Sugar Company with the values given by the Wieninger and Kubadinow (1973) formula. The corrected sucrose, industrial loss and yield values were also calculated with the Wieninger and Kubadinow formulae.

Soil properties were determined after harvest. In each plot, a composite soil sample was obtained at three different point in the crop row in each subplot using an auger (Ø 5 cm), mixed and placed in labeled bags. Texture, pH, conductivity and organic matter content were then determined using standard methods (MAPA., 1994).

Levels of available phosphorus, exchangeable sodium and magnesium and soluble boron were also determined (Table 1).

**TABLE 1:** Soil parameters measured at the end of the sugar beet productive cycle.

	Sand (%)	Silt (%)	Clay (%)	Texture	pH	Conductivity (mmhos/cm)	Organic matter (%)	Phosphorus, P (ppm)	Potassium, K (ppm)
ST1	43.84	32	24.16	Loam	8.3	0.44	2.05	50	251
ST2	39.84	34	26.16	Loam	8.2	0.5	1.9	51	177
ST3	49.84	24	26.16	Loamy-clay-sand	8.5	0.5	1.94	49	409
ST4	49.84	28	22.16	Loam	8.3	0.43	1.82	47	265
TB1	47.84	30	22.16	Loam	8.3	0.84	1.74	54	281
TB2	45.84	28	26.16	Loam	8.5	0.46	1.74	50	276
TB3	43.84	32	24.16	Loam	8.2	0.55	1.78	46	283
TB4	39.84	34	26.16	Loam	8.3	0.48	2.32	45	247
TT1	39.84	34	26.16	Loam	8.2	0.44	1.59	53	276
TT2	41.84	30	28.16	Loamy-clay	8.3	0.49	1.59	45	246
TT3	43.84	28	28.16	Loamy-clay	8.3	0.47	1.86	41	354
TT4	41.84	30	28.16	Loamy-clay	8.3	0.48	2.01	46	355

	Magnesium, Mg (ppm)	Carbonates (%)	Active lime (%)	Exchangeable calcium (ppm)	Exchangeable sodium (ppm)	Boron, B (ppm)
ST1	496	0.15	x	x	468	0.95
ST2	583	0.23	x	x	373	0.88
ST3	510	0.38	x	x	495	0.95
ST4	503	0.31	x	x	206	0.75
TB1	523	0.31	x	x	420	0.88
TB2	542	0.15	x	x	398	0.83
TB3	598	0.23	x	x	493	0
TB4	561	0.38	x	x	300	0.83
TT1	501	0.31	x	x	323	0.83
TT2	537	0.15	x	x	323	1.2
TT3	581	0.23	x	x	233	1.13
TT4	630	0.31	x	x	232	1

*Six applications of a mixed PGPRs culture (Pseudomonas fluorescens Pf0-1 and P. chlororaphis CECT 462) on the productivity of sugar beet were performed. Three different treatments, with four replicates, were performed: TB, without seed coating and with bacterial inoculum application; TT, with seed coating and ST, without seed coating in a completely randomized block design.*

### Photosynthesis Parameters Measurement

The photosynthetic status of a plant can be used as an indicator of its physiological status with respect to biostimulation or after a pathogenic attack. Foliar pathogens can cause the reduction of photosynthetic active leaf area, because of the leaf damage and the disturbance of photosynthesis in the remaining or surrounding leaf area (Berger et al., 2004; Robert et al., 2006). For instance, when a CLS disease severity on sugar beet of reached 3–6%, photosynthesis is reduced (Levall and Bornman, 2000). Thus, photosynthesis was measured in 10 leaves from 10 healthy plants with similar vegetative state in each subplot, 2 days after the third PGPRs inoculation (03 July 2018). This date was chosen because the physiological status of the plants was at the highest stage of sugar production.

A portable FMS2 fluorimeter (Fluorescence Monitoring System, Hansatech, Norfolk, United Kingdom) was used to measure fluorescence emission of chlorophyll in leaves previously adapted to darkness, to determine the efficiency of photosynthesis and to diagnose the presence of stress factors that decrease it (Krause and Weis, 1991; Baker, 2008). It also has the advantage of being a non-destructive technique. Two consecutive measurements were performed on the same leaf. The first one, corresponding to minimal fluorescence (Fo), is taken with the leaf adapted to dark conditions using a clamp for 20 min; thereafter the measure was repeated after a saturating light pulse, corresponding to the maximum fluorescence (Fm). With these two parameters, we calculated the maximum quantum yield of photosystem II (PSII) that indicates the maximum amount of energy that PSII could potentially expend in photochemical processes, which is calculated as Fv/Fm, where Fv is variable fluorescence = Fm − Fo. In the second step, we measured fluorescence emitted by the leaves adapted to light (Fs), and fluorescence when subjected to a saturating light pulse (Fm′ = maximum fluorescence measured in a state adapted to light). These parameters allowed us to calculate the quantum yield of PSII (ΦPSII), as ΦPSII = (Fm′ − Fs)/Fm′, and to quantify the proportion of energy absorbed by PSII that is used in photosynthetic electron transport (Genty et al., 1989), which therefore reveals the actual amount of energy that may be used for photochemical processes. Finally, we calculated NPQ (non-photochemical quenching) parameter. It was calculated as NPQ = (Fm − Fm′)/Fm′, a parametric indicator of the proportion of energy received that is dissipated as heat and therefore not used for photochemical processes (Ögren and Baker, 1985; Baker, 2008; Rodríguez-Moreno et al., 2008). All data were processed with MODFL2 software.

### Determination of Resistance to Plant Pathogens

The incidence of *Cercospora beticola* and *Erysiphe betae* was recorded throughout the production cycle at four timepoints: after third, fifth, and six PGPRs spraying applications and after the final harvest. Visual assessment of diseases was scored on a four-point scale, where 1 = 0–25% of the replicated area that was affected, 2 = 26–50% of the replicated area that was affected, 3 = 51–75%, and 4 = 76–100%, for each fungal pathogen (Supplementary Figure 2).

Infection index was calculated as the percentage of affected plants in each replicate as Index (%) = N/N_t_, where N is the number of affected plants in each replicate and N_t_ is the total number of plants per replicate.

Severity was determined at pathogen assessment-time 2 of the production cycle, the day on which both fungal pathogens reached their highest growth. The severity index was calculated as the percentage of affected leaves on a randomly selected sugar beet plant. The sugar beet plant selected in each replicate was a representative plant of medium size located at the center of the plot. Severity (%) = L/L_t_, where L is the number of affected leaves and L_t_ the total number of leaves. The visual assessment was evaluated on all four timepoints, the infection index was evaluated at timepoints 2 and 3, and the severity index, at timepoints 2.

### Statistical Analysis

One-way analysis of variance (ANOVA) using treatment as fixed factor was performed after checking for normality and homogeneity of variances with Kolmogorof-Smirnof’s and Levene’s test, respectively, LSD test was used to calculate significative differences between treatments. These analyses were carried out using STATGRAPHICS Plus 4.0 software. One and two-ways ANOVA was performed to evaluate the differences between treatments in each fungal disease and between sampling days.

## Results

### Sugar Beet: Crop Production Yield and Quality

Percentage plant survival rates per replicate were: 92, 96, and 98% for the ST, the TB and the TT assays, respectively. The plant populations of the three assays were very similar and there were no significant differences between the ST, the TB, and the TT plant populations. Sugar beet yield and quality measurement were noted at the end of the production cycle. Table 2 shows the biometric parameters reported from the three treatments (TB, ST, and TT). Both the total biomass (plant weight) and the sugar content of the beets from the TB plots were significantly higher than those for the other treatments. There were no significant differences between those values for the ST and the TT treatments, although the values of the ST treatment were somewhat higher (Table 2). Root weight and maximum beet perimeter values of the beets given the TB treatment were significantly higher than for the ST and the TT treatments, between which there was no significant variation. The highest aerial biomass and root length values were found in the beets given the TB treatment, although differences with regard to beets given the other two treatments were not significant (*p* ≥ 0.05) (Supplementary Figures 3, 4). Significant differences were found for both corrected and total sucrose content (polarization) values between TB treatment (with the lowest value) and the other two and no significant differences were found between the latter two (ST and TT) (Table 2). The results of soil parameters (edaphic characteristics), at the end of the productive cycle (upon harvest), showed no significant differences between the ST, the TT and the TB treatments.

**TABLE 2:** Sugar beet physiological parameters measured at the end of the productive cycle.

Parameters	ST	TB	TT
Total biomass (kg)	17.55 ± 0.99^a^	20.35 ± 1.47^b^	13.85 ± 1.02^a^
Sugar (kg/10 plants)	2.30 ± 0.17^a^	2.73 ± 0.22^b^	2.61 ± 0.18^a^
Root biomass (g/plant)	1414.17 ± 113.71^a^	1694.17 ± 73.14^b^	1164.17 ± 86.77^a^
Maximum beet			
Perimeter (cm/plant)	44.42 ± 1.47^a^	52.02 ± 1.35^b^	41.92 ± 1.24^a^
Polarization (%/plant)	17.83 ± 0.12^a^	16.78 ± 0.25^b^	18.15 ± 0.08^a^
Corrected sucrose (%/plant)	15.11 ± 0.17^a^	13.79 ± 0.28^b^	15.80 ± 0.06^a^
N-amino	0.47 ± 0.07^a^	0.63 ± 0.11^a^	0.35 ± 0.05^a^
Potassium, K	5.60 ± 0.20^a^	5.68 ± 0.17^a^	5.05 ± 0.13^a^
Sodium, Na	1.34 ± 0.28^ab^	2.00 ± 0.28^a^	0.85 ± 0.11^b^
Industrial Loss%	15.23 ± 0.52^a^	17.80 ± 0.66^b^	12.92 ± 0.34^c^
Industrial Yield%	84.77 ± 0.52^a^	82.20 ± 0.66^b^	87.08 ± 0.34^c^

*Six applications of a mixed PGPRs culture (Pseudomonas fluorescens Pf0-1 and P. chlororaphis CECT 462) on the productivity of sugar beet were performed. Three different treatments, with four replicates, were performed: TB, without seed coating and with bacterial inoculum application; TT, with seed coating and ST, without seed coating in a completely randomized block design. A simple ANOVA was performed; letters show significant differences (p ≤ 0.05) between treatments. Values are means (10 replicates) ± S.E.*

### Photosynthesis Parameters Measurement

**FIGURE 1:**
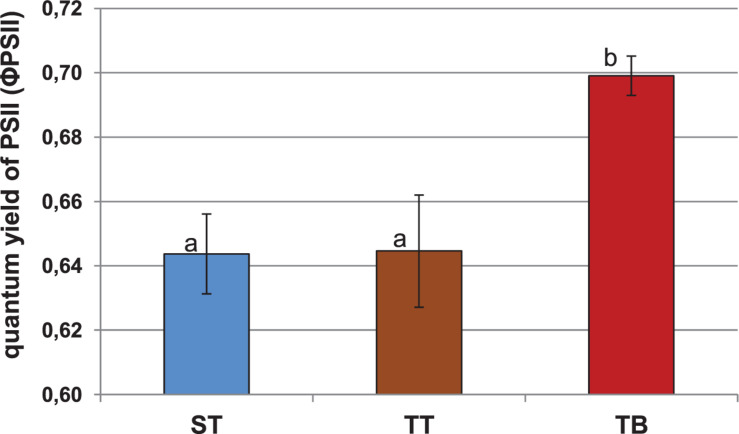
Quantum yield of photosystem II (ΦPSII) for 12-weeks-old sugar beet plants (2 days after the third PGPRs inoculation, 03 July 2018). Six applications of a mixed PGPRs culture (*Pseudomonas fluorescens* Pf-01 and *P. chlororaphis* CECT 462) on the productivity of sugar beet were performed. Three different treatments, with four replicates in a completely randomized block design, were performed: TB, without seed coating, with PGPRs inoculum application and chemical spraying; TT, with seed coating and chemical spraying and without PGPRs inoculum and ST, without seed coating, chemical spraying and PGPRs inoculum. A simple ANOVA was performed; letters show significant differences (*p* ≤ 0.05).

Figure 1 shows the quantum yield of PSII (ΦPSII). The beet showed significantly higher mean values (0.70) after the TB treatment than after the ST (0.64) and the TT (0.64) treatments. In relation to maximum quantum yields of photosystem II (PSII) and the non-photochemical quenching (NPQ) parameters, we observed no significant differences between either the ST, or the TT and the TB treatments (Supplementary Figures 5, 6).

### Determination of Resistance to Plant Pathogens

**FIGURE 2:**
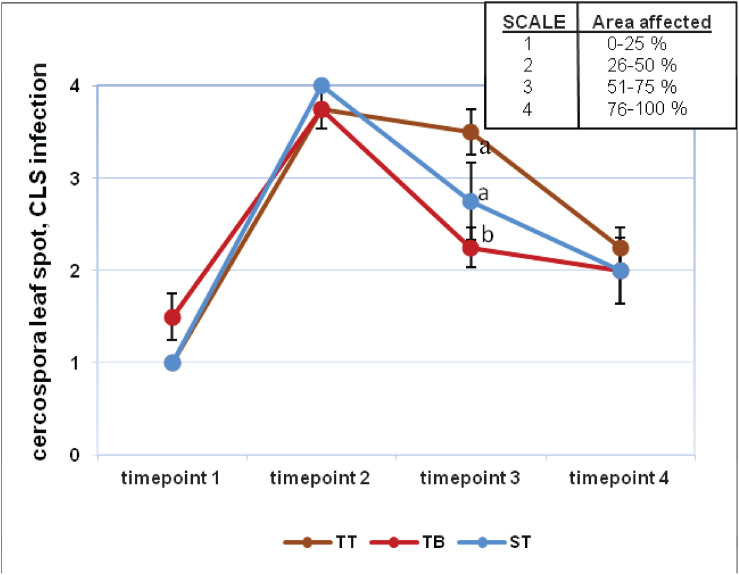
Visual infection evaluation (cercospora leaf spot, CLS) during the experiment at four timepoints. Fungal diseases were scored on a four-point scale (1–4). Six applications of a mixed PGPRs culture (*Pseudomonas fluorescens* Pf-01 and *P. chlororaphis* CECT 462) on the productivity of sugar beet were performed. Three different treatments, with four replicates in a completely randomized block design, were performed: TB, without seed coating, with PGPRs inoculum application and chemical spraying; TT, with seed coating and chemical spraying and without PGPRs inoculum and ST, without seed coating, chemical spraying and PGPRs inoculum. Two-way ANOVA was carried out for CLS infection values of three treatments along four evaluation times.

Visual assessment revealed that the evolution of *Erysiphe betae* (powdery mildew) and *Cercospora beticola* (cercospora) infection was similar for all three treatments (Supplementary Figure 7 and Figure 2). For powdery mildew, the highest degree of infection was found on timepoint 2, although it subsequently decreased progressively. All values from timepoint 2 were above 3 points on the established 4 point-scale. The values from timepoints 1 and 3 were very similar and yet very different with respect to timepoints 2 and 4 (Supplementary Figure 7). The results for cercospora infection were similar to those for mildew, although the values obtained over the four evaluated timepoints differed greatly between each other. All values from timepoint 2 were very near to 4 points on the established 4 point-scale. On timepoint 3, the highest degree of cercospora infection was found in the TT treatment (3.5), whereas the values for ST and TB were 2.75 and 2.25, respectively (Figure 2). The index values for these two pathogens on timepoints 2 and 3 did not differ significantly between the three treatments (Supplementary Figures 8A,B). The degree of infection was higher on timepoint 2, and this value differed significantly from that found for timepoint 3. On timepoint 3, the highest index for mildew infection was found for the TB treatment (41.33%), while the ST and the TT treatments gave values of 22.31 and 12.42%, respectively (Supplementary Figure 8A). In contrast, on timepoint 3, the TT treatment had the highest cercospora infection index at 67.45%, while the ST and the TB treatments had 59.37 and 56.37%, respectively (Supplementary Figure 8B). The mean severity for mildew and cercospora did not differ significantly between any of the three treatments (Supplementary Figure 9), although a significant difference in the severity of these two pathogens was found for the ST treatment (Supplementary Figure 9).

## Discussion

The present work has studied the effect of PGPRs inoculation of sugar beet on agronomic performance, photosynthetic process changes and biocontrol effects on two main fungal pathogens.

In the 2018 campaign, the Sociedad Cooperativa General Agropecuaria (ACOR) received 3,183,974 tons of sugar beet, with a mean purity of 17.50%, from the north of the region of Spain. The results of our treatments fall within the purity interval obtained by the ACOR, with values ranging between 16 and 18.4%. The mean purity obtained with the PGPRs inoculation assays (TB treatment) was 16.77%, a value close to that obtained by the ACOR (Table 2). The beets given the TB treatment had the highest sugar levels, total biomass, root biomass, maximum beet perimeter, N-amino, Potassium and Sodium content values. In all cases, those same values following the ST treatment are at an intermediate point between those for the TB and the TT treatments (Table 2). From these results, PGPRs inoculations appear to enhance the previously discussed biometric parameters of sugar beet plants. The PGPR strains applied in the present experiment belong to *Pseudomonas* genus, one of the most effective phosphate solubilizing bacteria and is considered as optimal specie for their stable P-solubilizing effects (Huang et al., 2010). Therefore, this biostimulant effect could be explained by the ability of the applied PGPR strains to solubilize phosphate. This mobilization of insoluble soil phosphate into bioavailable forms that can be taken up by the plant root (Monds et al., 2006). Previous related field trials have also reported significantly increased production yields for sugar beet crops upon application of various microorganisms (Cakmakci et al., 2001). Indeed, this latter study reported increases in sugar beet root production of between 6.1 and 13%, with an increase in sugar content of between 2.3 and 7.8%, in plants inoculated with *Bacillus polymyxa*, *Burkholderia* sp. and *Pseudomonas* sp. From the results of our experiment, we suggest that the two assayed PGPRs triggered growth promotion in the treated sugar beet plants. This PGPRs-induced plant growth was also reported in similar studies, with plants treated with these strains growing taller and more vigorously (Suslow and Schroth, 1982; Nandakumar et al., 2001). Bakker et al. (2020) have reviewed in depth the root-associated microbiota and their functions in plant health and especially on how modern microbiomics technologies can help to decipher complex processes that govern the assembly and functioning of the root microbiome.

Nowadays, the use of photosynthetic parameters as metabolic markers of systemic induction by bacterial agents is increasing. Our results were similar to those obtained by Zou et al. (2005), who studied these variations in photosynthetic parameters. From our results, we could see that PGPRs inoculation (TB treatment) produced significant differences in the quantum yield of PSII (ΦPSII) (Figure 1). This parameter indicates the real energy that the plants are using in the photochemical processes, at any given time. It seems that PGPRs inoculation could exert a beneficial effect on promoting the physiological stage of sugar beet plants, with regard to the other treatments (ST and TT). These photosynthetic modifications induced by PGPRs have been confirmed by other authors (Van Loon et al., 1998). The NPQ values for the ST, the TT and the TB treatments were also very similar, which means that the three treatments have, *a priori*, the same energy loss at the measurement stage (Supplementary Figure 6). Normally, NPQ reduction is observed in plants subject to different stress conditions (Whalen et al., 1991; Yamane et al., 2008). The results of the photosynthetic parameters provided evidence that PGPRs inoculation (TB treatment) showed the highest value for ΦPSII and the NPQ, the lowest value, even though the PSII was very similar in all treatments. Besides, the TT treatment showed a very low quantum yield of PSII, the highest NPQ value, and was the treatment with the highest amount of energy loss (Figure 1 and Supplementary Figure 6). The changes that occur in the photosynthetic parameters due to the use of PGPRs are not surprising because these PGPRs could be recognized as pathogen agents by plants and promote some plant-microorganisms interactions in relation to the Systemic Resistance Induced in sugar beet plants.

Although the results obtained with the biological control of cercospora and mildew were not the most successful for the TB treatment, they should nevertheless be closely analyzed, in order to design subsequent studies to perfect the application of PGPRs to sugar beet. Researchers at Montana State University (Bargabus et al., 2002, 2004) obtained a similar reduction of CLS in sugar beet plants applying a mixed microbial suspension. CLS and mildew infection indexes were higher on timepoint 2 with respect to timepoint 3 of the experiment for all the treatments (Supplementary Figure 8). In relation to the CLS infection index on timepoint 3, TB trials showed lower values than those obtained in TT and ST trials (Supplementary Figure 8B). The TB mean severity showed an intermediate value compared to the other treatments (Supplementary Figure 9). Bargabus et al. (2002, 2004) found that the application of *Bacillus pumilus* (strains 203-6 and 203-7) and *Bacillus mycoides* Bac J reduced the severity of CLS in sugar beet. We could conclude that *Cercospora beticola* infection was slightly lower in those PGPRs inoculated plants with respect to non-treated plants (timepoint 3) (Figure 2).

Therefore, co-inoculation of PGPR strains exerts a beneficial effect on sugar beet production, in such a way that physiological modifications inside the sugar beet plants increase its agricultural productivity. Qingxiao et al. (2016) have demonstrated that *B. velezensis* BAC03 can significantly enhance plant growth. Results showed that multiple applications of BAC03 were better than a single application in enhancing radish growth. This might be due to a combination of survival of the bacterium and prolonged period of maintaining the bacterial population at a high level by multiple applications. Similarly to our several PGPRs inoculations, in the research of Qingxiao et al. (2016), BAC03 was applied at five different timepoints during radish growth, including five days before planting (DBP), 1, 10, 20, and 30 DAP with the same concentration of 10^5^ CFU cm^3^ potting mix. Fresh and dry weight of leaves and roots were determined at harvest, 6 weeks after planting. Similar to our *Pseudomonas* inoculation, sugar beet seeds were treated with the mix bacterial suspensions for 30 min and also during sowing (Fikrettin et al., 2004).

Hence, PGPR efficacy depends on several factors, but it is assessed according to the specific PGPR strains that are used, the amount of inoculum (CFU/ml) and the plant inoculation method. As Munees and Mohammad (2011) indicated, the use of PGPR to augment crop productivity has been limited largely due to the variability and inconsistency of results observed under laboratory, greenhouse and field trials. Soil is an unpredictable environment and an intended result is sometimes difficult to achieve. Climatic variations has also a large impact on the effectiveness of PGPR but sometimes unfavorable growth conditions in the field are to be expected as a normal functioning of agriculture (Zaidi et al., 2009). Despite all these factors, there are many studies that prove the increase in crop yields following PGPR applications in the growth chambers and field trials (Munees and Mohammad, 2011).

The two assayed PGPR strains, *Pseudomonas fluorescens* Pf0-1 and *Pseudomonas chlororaphis* CECT 462, triggered a significant increase in sugar beet production yield and quality. Our results have shown that, on the whole, the beneficial effects of PGPRs are directly observable. There were increases of sugar beet physiological and photosynthetic parameters. Indirectly, PGPRs co-inoculation did not exert a desirable biocontrol against powdery mildew and cercospora infections.

Finally, PGPRs inoculation techniques used with different crops can be complemented with more traditional agricultural techniques, as far as may be required to ensure sustainable agricultural production.

## Data Availability Statement

The original contributions presented in the study are included in the article/Supplementary Material, further inquiries can be directed to the corresponding author/s.

## Author Contributions

All authors listed have made a substantial, direct and intellectual contribution to the work, and approved it for publication.

## Conflict of Interest

LM-B was employed by the company Syngenta. The remaining authors declare that the research was conducted in the absence of any commercial or financial relationships that could be construed as a potential conflict of interest.

## Footnotes

1 http://www.ebi.ac.uk/Tools/sss/fasta/nucleotide.html

## Supplementary Material

Supplementary Figure 1 Timelines for ST, TB and TT treatments. Six applications of a mixed PGPRs culture (*Pseudomonas fluorescens* Pf-01 and *P. chlororaphis* CECT 462) on the productivity of sugar beet were performed. Three different treatments, with four replicates in a completely randomized block design, were performed: TB, without seed coating, with PGPRs inoculum application and chemical spraying; TT, with seed coating and chemical spraying and without PGPRs inoculum and ST, without seed coating, chemical spraying and PGPRs inoculum.

Supplementary Figure 2 Evaluation of the incidence of **(A)** Cercospora Leaf Spot, CLS and **(B)** Powdery Mildew throughout the productive cycle of sugar beet. The fungal incidences of both pathogens were scored on a four-point scale. For the assessment of fungal incidences, the following parameters were recorded: for CLS incidence, extent of necrotic leaf area with characteristic brown round and necrotic spots and for Powdery Mildew, extent of white dust-like mycelium on leaf surface. Both fungal incidences were rated on a 1–4 scale according to the extent of the affected leaf area (1: between 0 and 25%, 2: between 26 and 50%, 3: between 51 and 75% and 4: between 76 and 100%).

Supplementary Figure 3 Aerial biomass measured at the end of the sugar beet productive cycle. Six applications of a mixed PGPRs culture (*Pseudomonas fluorescens* Pf-01 and *P. chlororaphis* CECT 462) on the productivity of sugar beet were performed. Three different treatments, with four replicates in a completely randomized block design, were performed: TB, without seed coating, with PGPRs inoculum application and chemical spraying; TT, with seed coating and chemical spraying and without PGPRs inoculum and ST, without seed coating, chemical spraying and PGPRs inoculum. A simple ANOVA was performed; letters show significant differences (*p* ≤ 0.05).

Supplementary Figure 4 Root length measured at the end of the sugar beet productive cycle. Six applications of a mixed PGPRs culture (*Pseudomonas fluorescens* Pf-01 and *P. chlororaphis* CECT 462) on the productivity of sugar beet were performed. Three different treatments, with four replicates in a completely randomized block design, were performed: TB, without seed coating, with PGPRs inoculum application and chemical spraying; TT, with seed coating and chemical spraying and without PGPRs inoculum and ST, without seed coating, chemical spraying and PGPRs inoculum. A simple ANOVA was performed; letters show significant differences (*p* ≤ 0.05).

Supplementary Figure 5 Maximum quantum yield of photosystem II (PSII) calculated as Fv/Fm for 12-weeks-old sugar beet plants (2 days after the third PGPRs inoculation, 03 July 2018). Three different treatments, with four replicates in a completely randomized block design, were performed: TB, without seed coating, with PGPRs inoculum application and chemical spraying; TT, with seed coating and chemical spraying and without PGPRs inoculum and ST, without seed coating, chemical spraying and PGPRs inoculum. A simple ANOVA was performed; letters show significant differences (*p* ≤ 0.05).

Supplementary Figure 6 Non-photochemical quenching (NPQ) for 12-weeks-old sugar beet plants (2 days after the third PGPRs inoculation, 03 July 2018). Three different treatments, with four replicates in a completely randomized block design, were performed: TB, without seed coating, with PGPRs inoculum application and chemical spraying; TT, with seed coating and chemical spraying and without PGPRs inoculum and ST, without seed coating, chemical spraying and PGPRs inoculum. A simple ANOVA was performed; letters show significant differences (*p* ≤ 0.05).

Supplementary Figure 7 Visual infection evaluation (powdery mildew) during the experiment at four timepoints. Six applications of a mixed PGPRs culture (*Pseudomonas fluorescens* Pf-01 and *P. chlororaphis* CECT 462) on the productivity of sugar beet were performed. Three different treatments, with four replicates in a completely randomized block design, were performed: TB, without seed coating, with PGPRs inoculum application and chemical spraying; TT, with seed coating and chemical spraying and without PGPRs inoculum and ST, without seed coating, chemical spraying and PGPRs inoculum. Two-way ANOVA was performed; letters show significant differences (*p* ≤ 0.05). ST, TB, and TT treatments showed no significant differences (same letters are not shown).

Supplementary Figure 8 Evaluation of the infection index, (a) powdery mildew and (b) cercospora leaf spot, CLS on timepoints 2 and 3 of the experiment. Six applications of a mixed PGPRs culture (*Pseudomonas fluorescens* Pf-01 and *P. chlororaphis* CECT 462) on the productivity of sugar beet were performed. Three different treatments, with four replicates in a completely randomized block design, were performed: TB, without seed coating, with PGPRs inoculum application and chemical spraying; TT, with seed coating and chemical spraying and without PGPRs inoculum and ST, without seed coating, chemical spraying and PGPRs inoculum. A simple ANOVA was performed; different capital letters for treatments and lowercase letters for time show significant differences (*p* ≤ 0.05). In CLS infection, ST, TB and TT treatments showed no significant differences (same letters are not shown). Vertical error bars are S.E.D.

Supplementary Figure 9 Evaluation of the severity of infection of powdery mildew and cercospora leaf spot (CLS) on timepoint two of the experiment. Six applications of a mixed PGPRs culture (*Pseudomonas fluorescens* Pf-01 and *P. chlororaphis* CECT 462) on the productivity of sugar beet were performed. Three different treatments, with four replicates in a completely randomized block design, were performed: TB, without seed coating, with PGPRs inoculum application and chemical spraying; TT, with seed coating and chemical spraying and without PGPRs inoculum and ST, without seed coating, chemical spraying and PGPRs inoculum. A simple ANOVA was performed between treatments; asterisk indicates significant differences between treatments (*p* ≤ 0.05). In powdery mildew, ST, TB, and TT treatments did not shown significant differences (same letters are not shown). Vertical error bars are S.E.D.
