# Supplementary material for: Microbial Inoculation for Productivity Improvements and Potential Biological Control in Sugar Beet Crops
Source: Front Plant Sci. 2020 Dec 22;11:604898. doi: 10.3389/fpls.2020.604898 (PMC7783361; doi:10.3389/fpls.2020.604898)
Supplement: Supplementary Figure 1 — Timelines for ST, TB and TT treatments. Six applications of a mixed PGPRs culture (Pseudomonas fluorescens Pf-01 and P. chlororaphis CECT 462) on the productivity of sugar beet were performed. Three different treatments, with four replicates in a completely randomized block design, were performed: TB, without seed coating, with PGPRs inoculum application and chemical spraying; TT, with seed coating and chemical spraying and without PGPRs inoculum and ST, without seed coating, chemical spraying and PGPRs inoculum. [file Data_Sheet_1.docx]

| 02 ^nd^ June 2018 | 17 ^th^ September 2018 | 08 ^th^ October 2018 | 26 ^th^ November 2018 |
| --- | --- | --- | --- |
| **timepoint 1** | **timepoint 2** | **timepoint 3** | **timepoint 4** |

**TT Treatment**

**SEEDING**

**RE-SOWING**

**CHEMICAL SPRAYING**

PHOTOSYNTHESIS MEASUREMENT

PATHOGEN ASSESSMENT

DAY 1

FINAL HARVEST

PATHOGEN ASSESSMENT

DAY 2

PATHOGEN ASSESSMENT

DAY 3

PATHOGEN ASSESSMENT

DAY 4

***23 days***

***61 days***

***30 days***

***33 days***

***36 days***

***49 days***

**TB Treatment**

**SEEDING**

**RE-SOWING**

PATHOGEN ASSESSMENT

DAY 1

FINAL HARVEST

PATHOGEN ASSESSMENT

DAY 2

PATHOGEN ASSESSMENT

DAY 4

***23 days***

***27 days***

***30 days***

***33 days***

***36 days***

***49 days***

**Fifth PGPRs spraying**

PHOTOSYNTHESIS MEASUREMENT

**Fourth PGPRs spraying**

**Third PGPRs spraying**

***22 days***

**CHEMICAL SPRAYING**

PATHOGEN ASSESSMENT

DAY 3

**Sixth PGPRs spraying**

***12***

***days***

**First PGPRs spraying**

**Second PGPRs spraying**

**ST Treatment**

**SEEDING**

**RE-SOWING**

PHOTOSYNTHESIS MEASUREMENT

PATHOGEN ASSESSMENT

DAY 1

FINAL HARVEST

PATHOGEN ASSESSMENT

DAY 2

PATHOGEN ASSESSMENT

DAY 3

PATHOGEN ASSESSMENT

DAY 4

***23 days***

***61 days***

***30 days***

***33 days***

***36 days***

***49 days***

**SUPPLEMENTARY FIGURE** **1** Timelines for ST, TB and TT treatments. Six applications of a mixed PGPRs culture (*Pseudomonas fluorescens* Pf-01 and *P. chlororaphis* CECT 462) on the productivity of sugar beet were performed. Three different treatments, with four replicates in a completely randomized block design, were performed: TB, without seed coating, with PGPRs inoculum application and chemical spraying; TT, with seed coating and chemical spraying and without PGPRs inoculum and ST, without seed coating, chemical spraying and PGPRs inoculum.

**(A)**

(1)

(2)

(3)

(4)


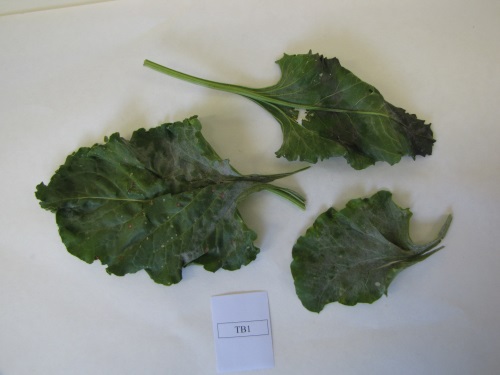

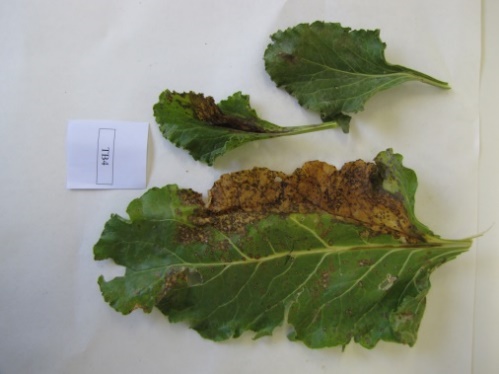


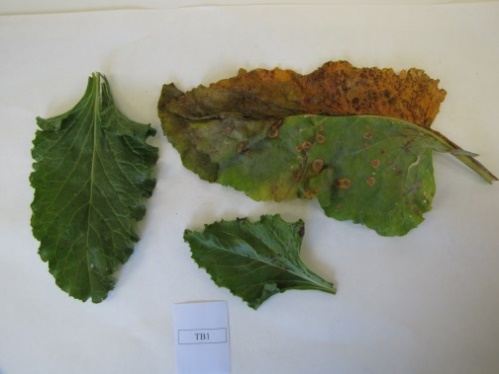

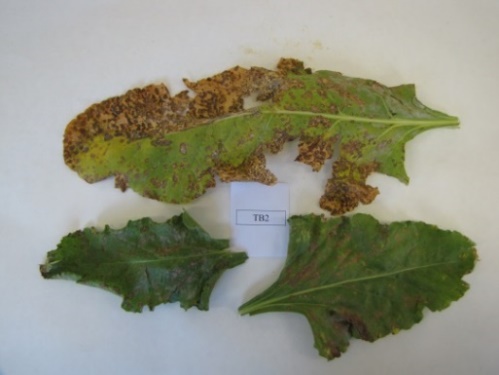


**(B)**

(1)

(2)

(3)

(4)


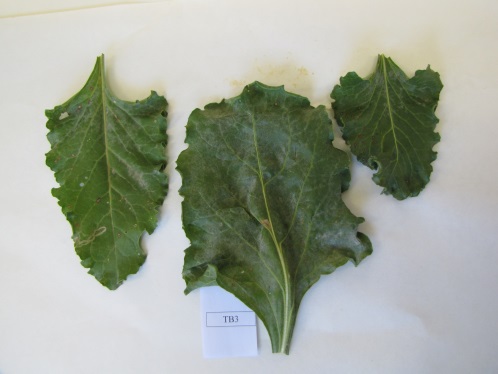

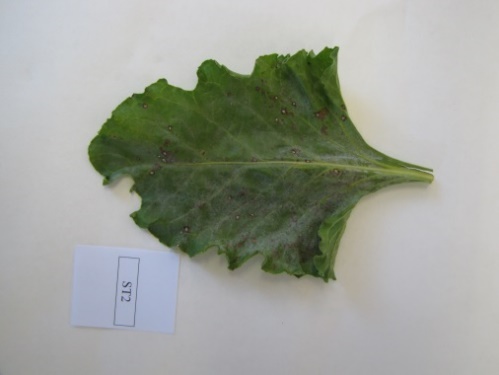


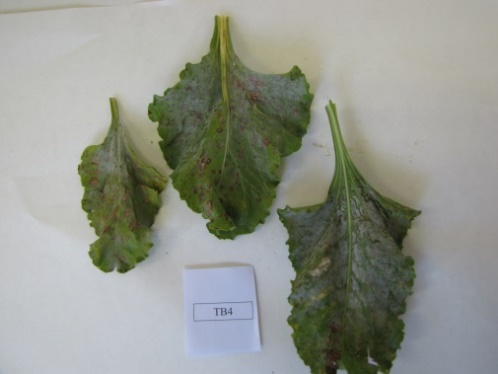

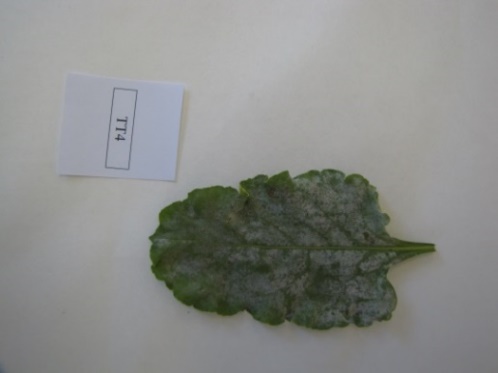


**SUPPLEMENTARY FIGURE 2.**  Evaluation of the incidence of (A) Cercospora Leaf Spot, CLS and (B) Powdery Mildew throughout the productive cycle of sugar beet. The fungal incidences of both pathogens were scored on a four-point scale. For the assessment of fungal incidences, the following parameters were recorded: for CLS incidence, extent of necrotic leaf area with characteristic brown round and necrotic spots and for Powdery Mildew, extent of white dust-like mycelium on leaf surface. Both fungal incidences were rated on a 1–4 scale according to the extent of the affected leaf area (1: between 0 and 25%, 2: between 26-50%, 3: between 51-75% and 4: between 76-100%).

**SUPPLEMENTARY FIGURE** **3**  Aerial biomass measured at the end of the sugar beet productive cycle. Six applications of a mixed PGPRs culture (*Pseudomonas fluorescens* Pf-01 and *P. chlororaphis* CECT 462) on the productivity of sugar beet were performed. Three different treatments, with four replicates in a completely randomized block design, were performed: TB, without seed coating, with PGPRs inoculum application and chemical spraying; TT, with seed coating and chemical spraying and without PGPRs inoculum and ST, without seed coating, chemical spraying and PGPRs inoculum. A simple ANOVA was performed; letters show significant differences (p≤0.05).

**SUPPLEMENTARY FIGURE** **4**  Root length measured at the end of the sugar beet productive cycle. Six applications of a mixed PGPRs culture (*Pseudomonas fluorescens* Pf-01 and *P. chlororaphis* CECT 462) on the productivity of sugar beet were performed. Three different treatments, with four replicates in a completely randomized block design, were performed: TB, without seed coating, with PGPRs inoculum application and chemical spraying; TT, with seed coating and chemical spraying and without PGPRs inoculum and ST, without seed coating, chemical spraying and PGPRs inoculum. A simple ANOVA was performed; letters show significant differences (p≤0.05).

**SUPPLEMENTARY FIGURE** **5** Maximum quantum yield of photosystem II (PSII) calculated as Fv/Fm for 12-week-old sugar beet plants (two days after the third PGPRs inoculation, 03 July 2018).Three different treatments, with four replicates in a completely randomized block design, were performed: TB, without seed coating, with PGPRs inoculum application and chemical spraying; TT, with seed coating and chemical spraying and without PGPRs inoculum and ST, without seed coating, chemical spraying and PGPRs inoculum. A simple ANOVA was performed; letters show significant differences (p≤0.05).

**SUPPLEMENTARY FIGURE** **6** Non-photochemical quenching (NPQ) for 12-week-old sugar beet plants (two days after the third PGPRs inoculation, 03 July 2018). Three different treatments, with four replicates in a completely randomized block design, were performed: TB, without seed coating, with PGPRs inoculum application and chemical spraying; TT, with seed coating and chemical spraying and without PGPRs inoculum and ST, without seed coating, chemical spraying and PGPRs inoculum. A simple ANOVA was performed; letters show significant differences (p≤0.05).

**SUPPLEMENTARY FIGURE** **7** Visual infection evaluation (powdery mildew) during the experiment at four timepoints. Six applications of a mixed PGPRs culture (*Pseudomonas fluorescens* Pf-01 and *P. chlororaphis* CECT 462) on the productivity of sugar beet were performed. Three different treatments, with four replicates in a completely randomized block design, were performed: TB, without seed coating, with PGPRs inoculum application and chemical spraying; TT, with seed coating and chemical spraying and without PGPRs inoculum and ST, without seed coating, chemical spraying and PGPRs inoculum. Two-way ANOVA was performed; letters show significant differences (p≤0.05). ST, TB and TT treatments showed no significant differences (same letters are not shown).

**SUPPLEMENTARY FIGURE** **8** Evaluation of the infection index, a) powdery mildew) and b) cercospora leaf spot, CLS on timepoints 2 and 3 of the experiment. Six applications of a mixed PGPRs culture (*Pseudomonas fluorescens* Pf-01 and *P. chlororaphis* CECT 462) on the productivity of sugar beet were performed. Three different treatments, with four replicates in a completely randomized block design, were performed: TB, without seed coating, with PGPRs inoculum application and chemical spraying; TT, with seed coating and chemical spraying and without PGPRs inoculum and ST, without seed coating, chemical spraying and PGPRs inoculum. A simple ANOVA was performed; different capital letters for treatments and lowercase letters for time show significant differences (p≤0.05). In CLS infection, ST, TB and TT treatments showed no significant differences (same letters are not shown). Vertical error bars are S.E.D.

**SUPPLEMENTARY FIGURE** **9**  Evaluation of the severity of infection of powdery mildew and cercospora leaf spot (CLS) on timepoint two of the experiment. Six applications of a mixed PGPRs culture (*Pseudomonas fluorescens* Pf-01 and *P. chlororaphis* CECT 462) on the productivity of sugar beet were performed. Three different treatments, with four replicates in a completely randomized block design, were performed: TB, without seed coating, with PGPRs inoculum application and chemical spraying; TT, with seed coating and chemical spraying and without PGPRs inoculum and ST, without seed coating, chemical spraying and PGPRs inoculum. A simple ANOVA was performed between treatments; asterisk indicates significant differences between treatments (p≤0.05). In powdery mildew, ST, TB and TT treatments did not shown significant differences (same letters are not shown). Vertical error bars are S.E.D.
